# Surgical treatment of chronic thromboembolic pulmonary hypertension in combination with a left anterior descending artery myocardial bridge: A case report

**DOI:** 10.3389/fcvm.2022.1061665

**Published:** 2022-12-09

**Authors:** Ilkhom K. Zugurov, Aleksei M. Osadchii, Maria A. Simakova, Olga M. Moiseeva, Mikhail L. Gordeev

**Affiliations:** ^1^Department of Cardiac Surgery, Almazov National Medical Research Centre, St. Petersburg, Russia; ^2^Department of Non-Coronary Heart Diseases, Almazov National Medical Research Centre, St. Petersburg, Russia

**Keywords:** chronic thromboembolic pulmonary hypertension, left anterior descending artery myocardial bridge, supracoronary myotomy, deep hypothermic circulatory arrest, pulmonary thromboendarterectomy

## Abstract

Pulmonary thromboendarterectomy is a potentially curative option for most patients with chronic thromboembolic pulmonary hypertension (CTEPH). However, a special group of patients with CTEPH requires simultaneous cardiac procedures. We report a rare case of successful surgical treatment of a CTEPH patient with a left anterior descending artery myocardial bridge. Despite the complexity of performing pulmonary thromboendarterectomy (PTE), the issue concerning the method of revascularization of the artery in the case of the left anterior descending artery myocardial bridge is controversial. PTE and supracoronary myotomy were performed. In our case, the optimal surgery method for the left anterior descending artery myocardial bridge was chosen intraoperatively based on the depth and length of the myocardial bridge. The patient's significant functional improvement after surgery and hemodynamic normalization were confirmed at the follow-up assessment. This case demonstrates rare but potentially dangerous pathologies that can be treated with minimal adverse effects.

## Introduction

Pulmonary thromboendarterectomy (PTE) is the gold standard for chronic thromboembolic pulmonary hypertension (CTEPH) patients' treatment; however, in some cases, combined heart surgery is required, which increases the intervention risk (1). The left anterior descending artery myocardial bridge (LAD-MB) is difficult to derive, and treatment options are still being discussed (2). LAD-MB management depends on the functional significance of coronary artery damage; however, its assessment is difficult in conditions of high pulmonary hypertension (2). The clinical case presented the experience of simultaneous pulmonary thrombendarterectomy and supra-arterial myotomy.

## Case report

**Figure 1:**
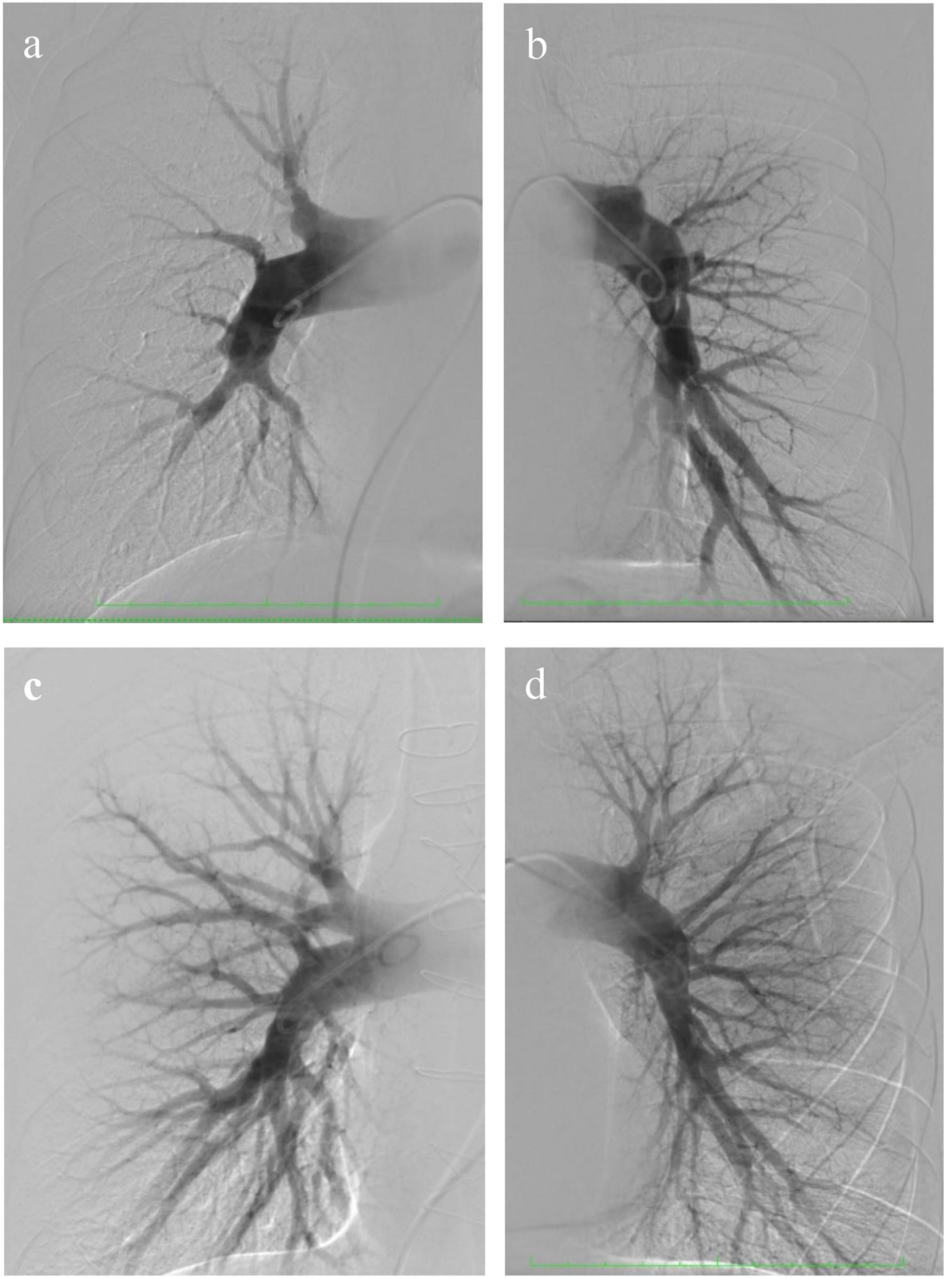
Pre- and post-operative selective pulmonary angiography. **(a)** Selective pulmonary angiography of the *right pulmonary* arteries identified: eccentric and extended stenoses in the segmental arteries A 1, 2, 4, 5, 6, 7, 8, and occlusion of A 3, 9, 10; **(b)** *left pulmonary artery:* occlusion of the upper lobe branch, roughness of the contours of the lower lobe branch, and eccentric and extended stenoses in the segmental arteries of the lingual segment and lower lobe; **(c,d)** selective pulmonary angiography after surgery.

**Figure 2:**
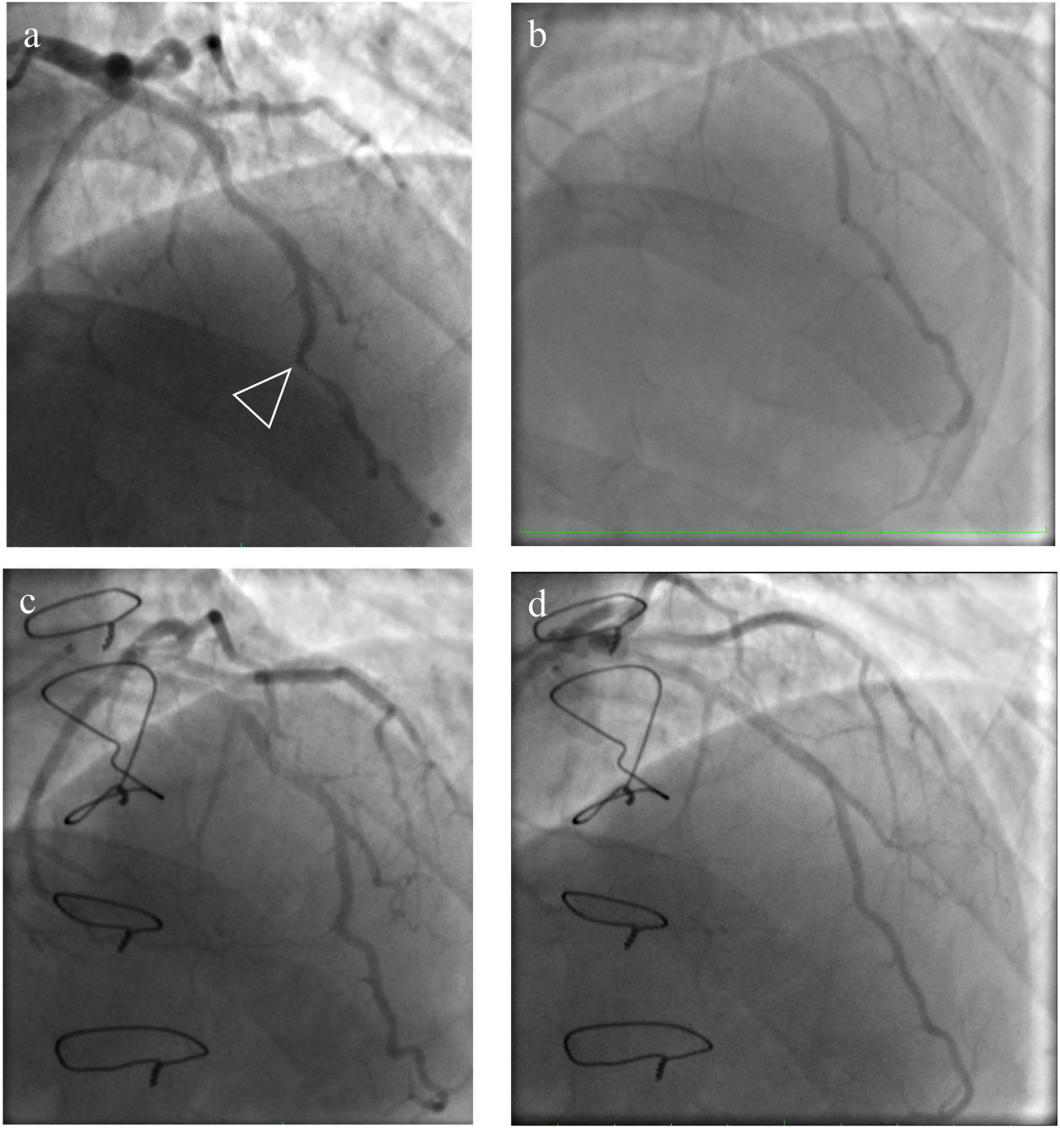
Pre- and post-operative view of coronary angiography. **(a)** An intramyocardially located section of the left anterior descending artery (LAD) in the middle third with stenosis in the systole up to 60–70%; **(c)** the systolic phase of the LAD-MB after the surgery; **(b,d)** the diastolic phase of the LAD-MB before and after the operation.

A 36-year-old man was referred to the Almazov National Medical Research Centre with a history of progressive heart failure (NYHA III functional class), chest pain, and the presence of severe pulmonary hypertension (PH) according to a transthoracic echocardiographic evaluation (TTE). The patient had a documented history of acute pulmonary embolism with a delayed diagnosis and initiation of anticoagulant therapy. TTE showed right atrial and right ventricle dilatation with preserved right ventricle function, left ventricle size, and function, and the estimated pulmonary artery systolic pressure was 120 mmHg (Table 1). Chest dual-energy CT-angiography and pulmonary angiography confirmed CTEPH (level II according to San Diego pulmonary endarterectomy disease levels) (Figures 1a,b). Right heart catheterization (RHC) defined precapillary PH: mean pulmonary artery pressure (PAP) of 59 mmHg, a pulmonary capillary wedge pressure (PCWP) of 13 mmHg, and a pulmonary vascular resistance of 7,98 Wood units. Riociguat treatment was started. Thrombophilia markers were tested, and antiphospholipid antibody syndrome was revealed as the main CTEPH risk factor. There were no data in favor of systemic lupus erythematosus or other systemic connective tissue diseases. Due to chest pain, selective coronary angiography was performed, and the LAD-MB was revealed. The length of the LAD-MD was ~25 mm, with left anterior descending artery stenosis in the systole reaching 70% (Figures 2a,c, arrowhead).

**Table 1:** Echocardiography parameters.

**Indicator**	**Before surgery**	**Post-surgery (7 days)**	**Post-surgery (8 months)**
RV (parasternal long axis size) (mm)	32	30	28
RV (four-chamber basal size) (mm)	52	48	36
TAPSE (mm)	17		24
PA pressure (systolic) (mmHg)	120	30	40
Tricuspid insufficiency (grade)	1	1	1
IVS/PW (mm)	14/11	13/11	10/9
EDS/ESS LV (mm)	48/30	47/32	48/32
EDV/ESV LV (ml)	108/34	100/40	107/41
EF (%)	68	60	64
LA (mm)	36	37	42

EDS, the end-diastolic size of the left ventricle; EDV, end-diastolic volume of the left ventricle; EF, ejection fraction of the left ventricle; ESV, the end-systolic volume of the left ventricle; ESS, the end-systolic size of the left ventricle; IVS, interventricular septum; LA, left atrium; PW, left ventricle posterior wall; RA, right atrium; RV, right ventricle; and TAPSE, tricuspid annular plane systolic excursion.

**Figure 3:**
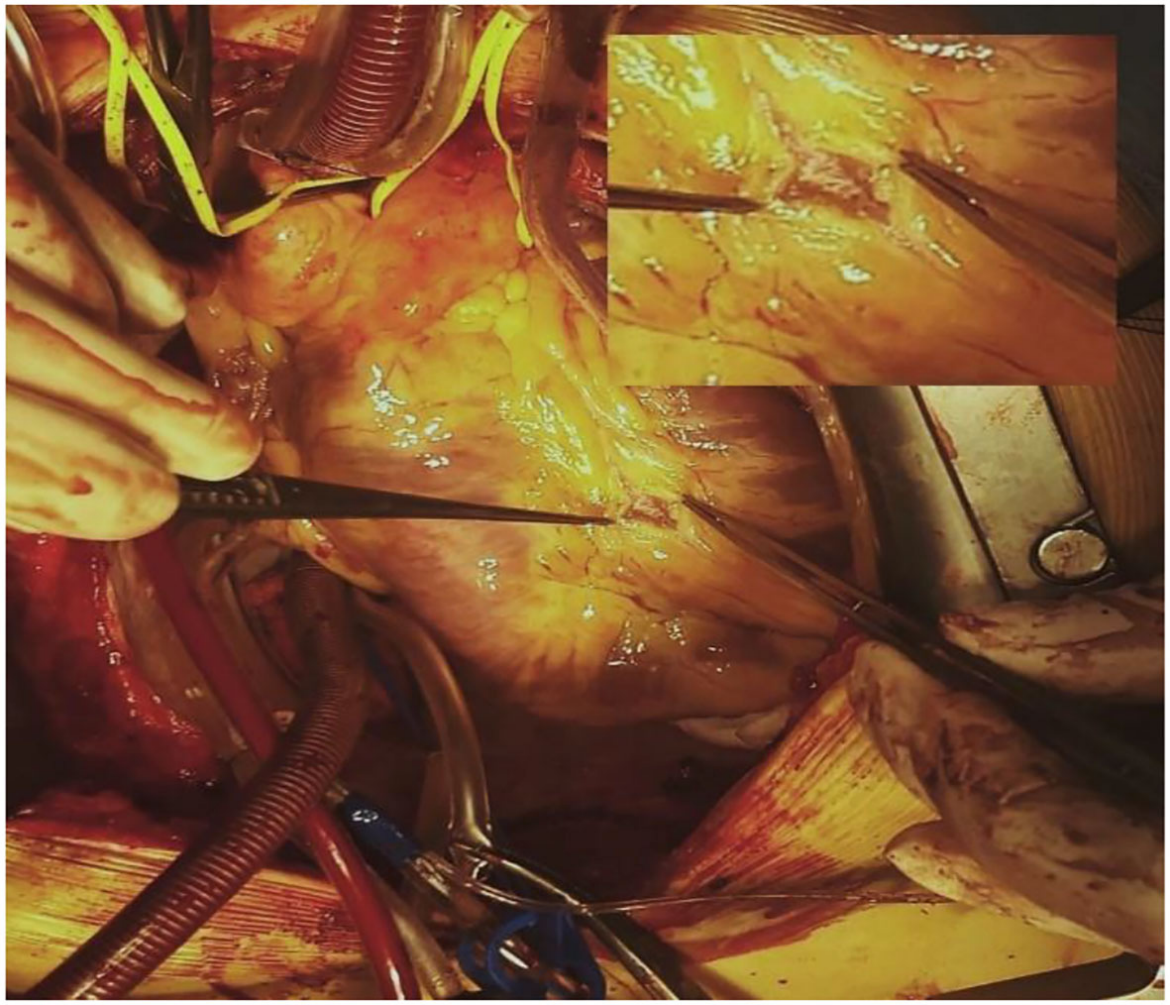
Intraoperative view of LAD-MB. The left anterior descending artery myocardial bridge lies at a depth of 4–5 mm with a length of 20–22 mm. LAD-MB was located on the border of the middle and distal thirds of the artery.

**Figure 4:**
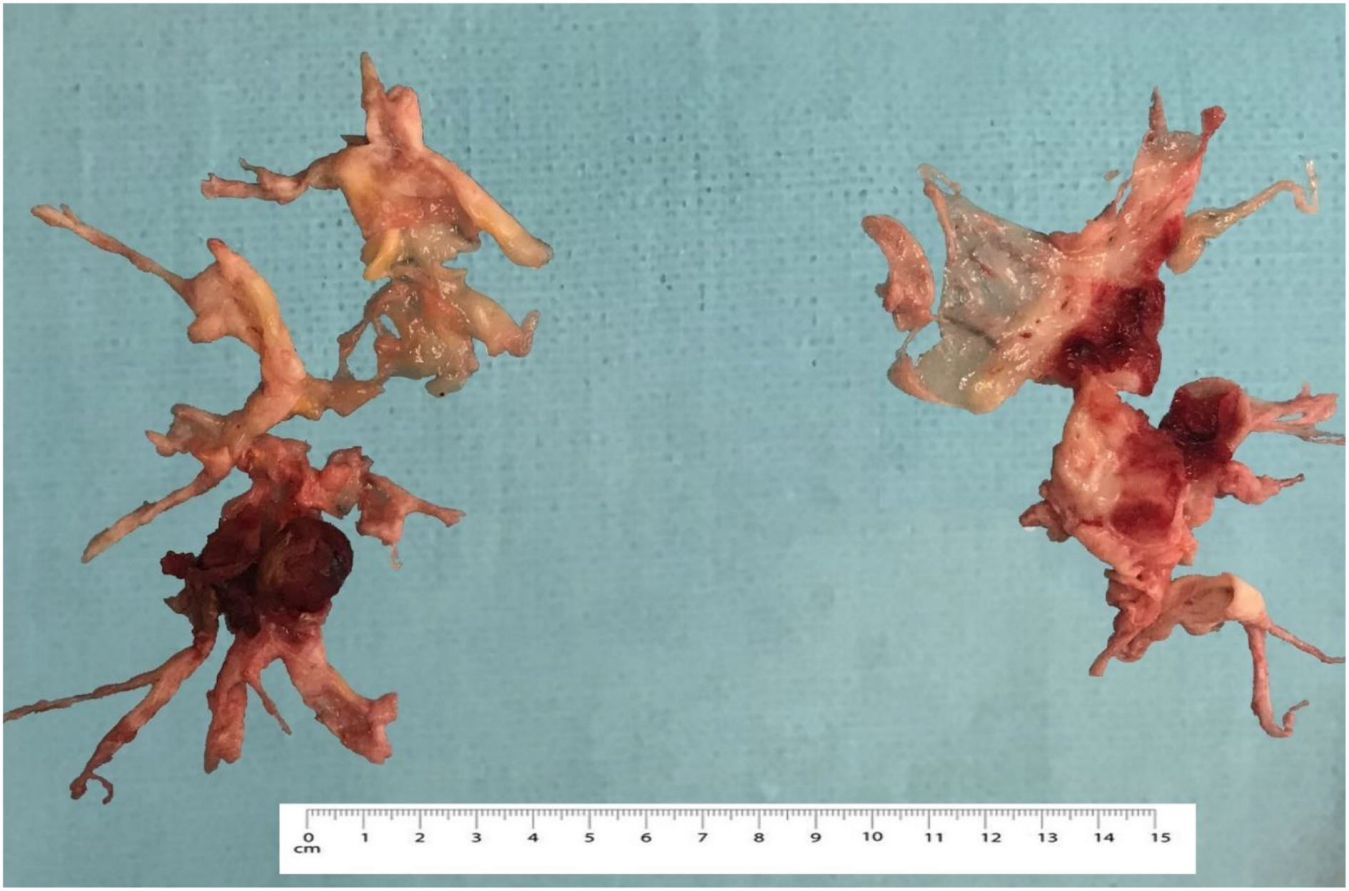
Intraoperative view of endarterectomized tissues.

Considering confirmed CTEPH with a level II lesion (San Diego classification of pulmonary endarterectomy disease levels) and a significant myocardial bridge of the left anterior descending artery, a pulmonary thromboendarterectomy in combination with a supracoronary myotomy was performed. Intraoperative revision showed LAD-MB lying at a depth of 4–5 mm for 20–22 mm (Figure 3), suggesting that a supra-arterial myotomy may be performed while the patient was being warmed up. Total surgery time was 330 min, and deep hypothermic circulatory arrest (DHCA) time was 73 min (four sessions were carried out). At the end of the operation, the mean pulmonary pressure had decreased by 65% and was 25 mmHg. The full view of endarterectomized tissues is shown in Figure 4.

The postoperative period was characterized by moderate cardiovascular and respiratory insufficiency without signs of reperfusion edema. The patient was extubated 22 h after the surgery. The intensive care unit treatment lasted 45 h without requiring PAH-targeted therapy. The patient was discharged on the 12th day after surgery with much dyspnea regression. Only vitamin K antagonists' treatment was prescribed.

The patient's health significantly improved and had no dyspnea at 8 months of follow-up. Selective pulmonary angiography demonstrated preserved blood flow over all pulmonary fields. No stenosis and occlusion of segmental branches were visible (Figures 1c,d). Coronary angiography found no local stenosis of the LAD artery (Figures 2c,d). The control RHC showed normalization of pulmonary hemodynamics with a reduced mean pulmonary artery pressure of 22 mmHg and an RVR of 1.7 WU (Table 2).

**Table 2:** Right heart catheterization parameters.

**Indicator**	**Before surgery**	**Post-surgery (1 day)**	**Post-surgery (8 months)**
Blood pressure (systolic/diastolic) (mmHg)	124/89	110/60	127/70
Pulmonary pressure (systolic/diastolic/mean) (mmHg)	102/31/59	31/10/20	32/10/22
PCWP (mmHg)	13	11	10
RAP (mmHg)	15	10	12
CO (per L/min)	5,76	5,9	7,1
CI (per L/min/m^2^)	2,45	2,5	3,02
PVR (Wood units)	7,98	2,0	1,7

CI, cardiac index; CO, cardiac output; PCWP, pulmonary capillary wedge pressure; PVR, pulmonary vascular resistance; and RAP, right atrium pressure.

In our case, the final diagnosis was chronic thromboembolic pulmonary hypertension with the third functional class according to the WHO classification of PH, CHD, and LAD-MB, with up to 70% stenosis in the systole. The surgical treatment was sternotomy, pulmonary endarterectomy, and simultaneous supracoronary myotomy of the LAD-MB.

## Discussion

This clinical case is a good example of a complicated course in PE. One of the well-known risk factors is the delay in PE diagnosis and, consequently, the late appointment of anticoagulants (more than 2 months from the PE clinic in our case). This factor is crucial for incomplete thrombi recanalization and their fibrosis transformation. Antiphospholipid syndrome is the second obvious CTEPH risk factor in this patient. Modern scientific research aims to discover new CTEPH formation predictors and study its pathogenesis. Such studies are important for optimizing the management of PE survivors.

Besides, our case demonstrates the complexity of the LAD-MB functional significance assessment in patients with CTEPH. Due to chest pain is a common complaint in such patients, it can be difficult to distinguish it from angina pectoris. It should be noted that stress echocardiography performance was limited by high pulmonary hypertension because the patient even could not perform any daily life activities. The pharmacological test to clarify the LAD-MB significance was limited by the riociguate treatment (adverse drug interactions). Additionally, nitroglycerin intracoronary administration for LAD-MB severity evaluation can sometimes increase artery narrowing in the bridge segment and vasodilate adjacent non-bridged coronary segments with myocardial ischemia occurrence (3).

In addition, Maeda and his colleagues observed the prevalence of plaque formation proximal to the bridged segment (4). Some reports showed that damaged segments stay free of plaques, likely due to the systolic compression of those segments, with subsequent improved lymphatic drainage and decreased lipid accumulation in those segments. Plaque formation in the artery upstream of the bridged segment has been frequently reported, especially after intravascular ultrasonography, which helped detect lesions not previously detected by angiography (5). This can be explained by endothelial injury associated with flow disturbances and wall shear stress at that proximal segment. Progression of proximal plaque formation is thought to increase with age, as evidenced in the study by Maeda and colleagues (4). Their study population was composed of patients aged 11–20 years, with a plaque burden of almost 19%, lower than that reported in adults (34%). Nonetheless, the presence of plaque in this young adult patient cohort with myocardial bridges is an important and concerning finding that may warrant a more aggressive approach in these cases.

Thereby LAD-MB potentially can be associated with acute coronary symptoms or with atherosclerotic disease progression. It is unknown whether patients with asymptomatic myocardial bridges should be medically treated or whether they should be exercise restricted for acute coronary event prevention. Further evaluation of myocardial bridges natural history may be necessary for these patients care and the adverse events occurance decrease (6).

A number of studies show that safe LAD-MB surgery can be considered if the narrowed segment of the artery lies at a depth of no more than 5 mm and a length of no more than 25 mm (7, 8). In our case, when assessing the narrowed segment intraoperatively, it was revealed that the bridge lies at a depth of 4–5 mm with a length of 20–22 mm. LAD-MB was located on the border of the middle and distal thirds of the artery. Furthermore, there was no artery narrowing in the LAD-MB segment in the diastole phase during coronary angiography before the operation.

All of the above arguments, combined with the procedure's technical feasibility, led us to decide on a combined cardiac surgery intervention for this CTEPH patient.

## Conclusion

Operable patients with CTEPH requiring combined heart surgery represent a special group with an increased risk of perioperative complications. The current case suggests that PTE combined with LAD-MB surgery is associated with acceptable perioperative morbidity and mortality rates and improved hemodynamic and functional status in the patient.

## Data availability statement

The original contributions presented in the study are included in the article/supplementary material, further inquiries can be directed to the corresponding authors.

## Ethics statement

The studies involving human participants were reviewed and approved by Extract No. 0801-21 from the protocol of the meeting of the Ethics Committee of the Almazov National Medical Research Center No. 01–21 dated January 18, 2021. The patients/participants provided their written informed consent to participate in this study.

## Author contributions

All authors listed have made a substantial, direct, and intellectual contribution to the work and approved it for publication.
